# Performance of T2Bacteria in relationship to blood cultures - a retrospective comparative study

**DOI:** 10.1007/s10096-024-04916-6

**Published:** 2024-08-03

**Authors:** David Yu, Anna Ekwall-Larson, Volkan Özenci

**Affiliations:** 1 Division of Clinical Microbiology, Department of Laboratory Medicine, Karolinska Institutet, SE-141 86 Stockholm, Hälsovägen, Stockholm, Sweden; 2 Functional Area of Perioperative Medicine and Intensive Care, Karolinska University Hospital, Stockholm, Sweden; 3 Department of Clinical Microbiology, Karolinska University Hospital, Stockholm, Sweden

**Keywords:** Bloodstream infection, Blood culture, T2, sepsis, Polymerase chain reaction, Rapid diagnostics

## Abstract

**Purpose:**

Blood culture (BC) is the gold standard for diagnosing blood stream infections (BSI) but is limited by long turnaround times (TAT) and low detection rate. The T2 Magnetic Resonance method (T2MR) offers a rapid, culture-independent alternative. The objective of this study was to compare the performance of the T2Bacteria assay to BCs in a real-world setting.

**Methods:**

Retrospective comparative study consisting of T2Bacteria samples and BCs sampled within 72 h from the T2Bacteria sample. The primary outcome was detections by BC and T2Bacteria, respectively. The secondary outcome was difference in TAT.

**Results:**

In total, 640 episodes were included, consisting of 640 T2Bacteria samples and 2,117 BCs. A median of three BCs was collected for each T2Bacteria sample. Overall positivity was 101 (15.8%) by either method. In 29 (28.7%) episodes, both T2Bacteria and BC were concordantly positive. In discordant episodes, 46/101 (45.5%) episodes were T2Bacteria positive/BC negative and 26/101 (25.7%) were T2Bacteria negative/BC positive (McNemar χ^2^, *p* < 0,05). In T2Bacteria positive/BC negative episodes, eight had growth of the same microorganism in a non-BC culture. Median (IQR) TAT for BC was 35 h and 30 min (25 h 50 min − 45 h 24 min), compared to 21 h and 3 min (17 h 6 min − 27 h 30 m) for T2Bacteria (*p* < 0.001), with longer delays for samplings occurring outside work hours.

**Conclusions:**

The study highlights a high discordance between T2Bacteria and BC and suggests complementary roles of the methods in BSI diagnostics. Furthermore, it is crucial to improve TAT by reducing preanalytical delays.

**Supplementary Information:**

The online version contains supplementary material available at 10.1007/s10096-024-04916-6.

## Background

Blood stream infection (BSI) and sepsis remain leading causes of mortality and morbidity worldwide [1]. Traditionally, blood cultures (BC) have been the gold standard in the microbiological diagnosis of BSI, providing identification of pathogens and antimicrobial susceptibility testing [2].

However, identification of microorganisms using BCs have several limitations, including reliance of time-consuming steps such as incubation and subculturing, which poses delays in targeting of the antibiotic treatment that can contribute to negative clinical outcomes [3]. Furthermore, the sensitivity of BCs can be compromised by various factors, such as prior antibiotic treatment, leading to inconclusive or misleading results. It has been shown that a significant proportion of BCs in sepsis is negative, in some studies reaching 30–40% [3, 4].

To overcome the limitation of BCs, there has been a growing interest in molecular diagnostic methods that can bypass BCs and identify microorganisms directly from blood samples [5–7]. One such emerging technology is the T2 Magnetic Resonance method (T2MR), a novel approach offering rapid diagnosis, initially designed for Candida spp. detection [8]. Briefly, T2MR first amplifies microbial DNA by PCR, after which probes enriched by superparamagnetic nanoparticles hybridize to the amplicon, enabling detection by the resulting change in the T2 signal of the sample [8]. T2MR was further developed to include a bacterial assay, T2Bacteria, with a panel that includes *Escherichia coli*,* Staphylococcus aureus*,* Klebsiella pneumoniae*,* Pseudomonas aeruginosa*,* Enterococcus faecium*,* and Acinetobacter baumannii* [9].

T2Bacteria may improve the diagnostic workflow compared to BC based diagnostics in BSI, most notably by the potential for increased speed and sensitivity [9]. The method also offers enhanced sensitivity, reportedly capable of detecting low colony-forming unit (CFU) levels that BCs often miss [8, 10]. Additionally, as T2Bacteria does not require a culture step, detection of bacteria is possible despite prior antibiotic treatment.

While T2Bacteria presents several promising features, real-world analytical performance data are still scarce. Furthermore, the performance of T2Bacteria must be placed in context with BCs, with potential complementary roles by each method. Most previous prospective studies are small [11–14] or have limited numbers of comparative BCs [9].

The objective of the present study was to investigate the real-world analytical performance of T2Bacteria panel in relationship to BCs and other relevant microbiological samples, in both community- and hospital-acquired infections. Specifically, the comparison was done against all BCs sampled around the time of T2Bacteria sampling. The primary outcomes were proportion of positive findings with T2Bacteria and the corresponding BCs, and the occurrence of discordant microorganisms detected by T2Bacteria and BCs. The secondary outcome was the turn-around time (TAT) for T2Bacteria and BCs.

## Methods

### Study design and setting

This was a retrospective comparative study comparing the performance of T2Bacteria panel against BCs for the diagnosis of BSI. The study period ranged from May 18, 2022, to November 21, 2023. The study was conducted in a single laboratory center, the Karolinska University Hospital Laboratory, which in turn receive microbiological samples from two tertiary care hospitals, four secondary care hospitals, and other care facilities including community and home care in the greater Stockholm area with a total of 6750 hospital beds [15].

### Inclusion and data collection

From here on, T2Bacteria will be referred to as T2. In Karolinska University Laboratory, T2 has been available since April 28, 2022, and indications for use is entirely determined by the treating physician at the suspicion of BSI. Data for all T2 samples during the study period were extracted from the hospital’s laboratory data system. In the present study, we defined an individual BSI episode as ranging from 72 h before, to 72 h after T2 sampling. We also explored the effect on included BCs by testing alternative time frames to ensure that the chosen time frame was appropriate. All BCs sampled during the episode were included for comparative analysis with the T2 sample. Inclusion of the T2 sample required at least one BC collected within 72 h from the T2 sampling. Multiple episodes from the same patient could be included unless the T2 sample in the episode had a preceding T2 sample within the last seven days. This was to ensure analysis of T2 samples from unique BSI episodes. Additionally, microbiological data for relevant clinical samples including urine, deep and superficial wounds, lower respiratory tract, cerebrospinal fluid, and non-blood sterile site samples were extracted from the laboratory data system. Data regarding age and gender of patients were collected as well as hospital ward (ICU or non-ICU) where the T2 sample was taken.

### Exclusion criteria

T2 samples were excluded if collected within 7 days after an included T2 sample from the same patient, keeping only the first T2 sample per episode for analysis. T2 samples were also excluded if the assay results were invalid. Additionally, T2 samples available from BSI episodes where no BCs were sampled were excluded, as it was not possible to compare T2 and BC data.

### T2 sampling procedure

T2 samples were collected according to the clinical protocol in place for T2Bacteria, which consisted of sampling four mL of blood in K_2_EDTA tubes. Samples containing less than three mL of blood were discarded. Samples arriving in the microbiology laboratory outside work hours were stored in the fridge at 2–8 °C for up to 72 h. The temperature of the samples was adjusted to room temperature prior to analysis. The analysis was performed using the on-site T2Dx^®^ (T2 Biosystems, Lexington, MA, USA) instrument, according to the manufacturer’s instructions.

### BC sampling procedure

The BC bottles were handled according to clinical routine at the Department of Clinical Microbiology, Karolinska University Hospital. The system used in the present study was BacT/ALERT Virtuo (Bio-Merieux, France). BCs were incubated until a positive signal was obtained, or for a duration of five days. For BCs that tested positive, Gram staining was performed, followed by subculturing onto agar plates. The colonies that subsequently grew on agar plates underwent species identification through matrix-assisted laser desorption/ionization time-of-flight mass spectrometry, provided by Bruker Daltonik in Bremen, Germany.

### Outcome measures

Number and proportions of episodes that were positive by BC and/or T2 was described. Common skin contaminants were not included in the analysis (Supplement Table S1). The performance of BCs was based on comparing the set of bacteria added from all BCs belonging to the episode, to the set of bacteria in the T2 sample. In the main comparison analysis, only bacteria included in the T2 panel were included. However, to study the impact of the limited repertoire of the T2 panel, we also performed a secondary analysis considering all microorganisms detected in BCs. The episodes were classified depending on the T2 and BC results as concordant (positive or negative) or discordant results. Discordant results were further classified depending on the outcome of the analyses. To investigate the significance of a T2 positive result in the setting of negative BCs, non-BC microbiological samples from the episode were analyzed. In absence of a reliable independent gold standard, adaptations of this approach have been used in several previous studies [9, 16, 17]. To validate this method, the same analysis was also performed for the T2 negative/BC positive group and the concordantly T2 positive/BC positive group.

The total TAT, defined in this study as the time from sampling to preliminary report, was divided into the time from sampling to arrival at the laboratory, and the time from arrival at the laboratory to preliminary report. For BC samples, the preliminary report was defined by the first reported identification of bacteria on the species level. For T2 samples, the preliminary report was the same as the final report and was defined by of the completion of reading the result of the T2 assay. TAT for T2 was assessed for differences in positive and negative samples, as well as by day of the week and time of the day of sampling.

### Statistical methods

Paired comparison for agreement between proportions of T2 and BC positivity was performed using McNemar’s χ^2^ test. Continuous variables were visually assessed for normality using a Q-Q plot, and comparisons were made with a two-sided t-test, or the Mann-Whitney U test, where appropriate. All statistical analyses were performed using Python 3.12.0 (modules scipy.stats 1.11.4, statsmodels.stats 0.14.0).

### Ethical considerations

In the present study, we exported sample data from the laboratory system, which did not contain sensitive personal information. The analysis was performed on the level of isolate data, and not on the patient level. As the data did not contain sensitive personal information, and the study was done on the information from the laboratory system and not the samples directly, this study did not require an ethical permit.

## Results

The study inclusion flow chart is found in Supplement Fig. 1. During the study period, in total 843 T2 samples were considered for analysis. We excluded 102 T2 samples as they were sampled less than 7 days after a previous T2 sample from the same patient. Two T2 samples were excluded due to invalid results of the assay. 99 T2 samples were further excluded as no associated BCs were found during the 72-hour time frame before and after the T2 sampling. Different scenarios for the choice of time frame are shown in Supplement Fig. 2.

After exclusion, 640 episodes from 544 patients were included, consisting of 640 T2 samples and 2,117 BCs in total. In 478/640 (74.7%) episodes, there was at least one non-BC microbiological sample collected.

**Fig. 1:**
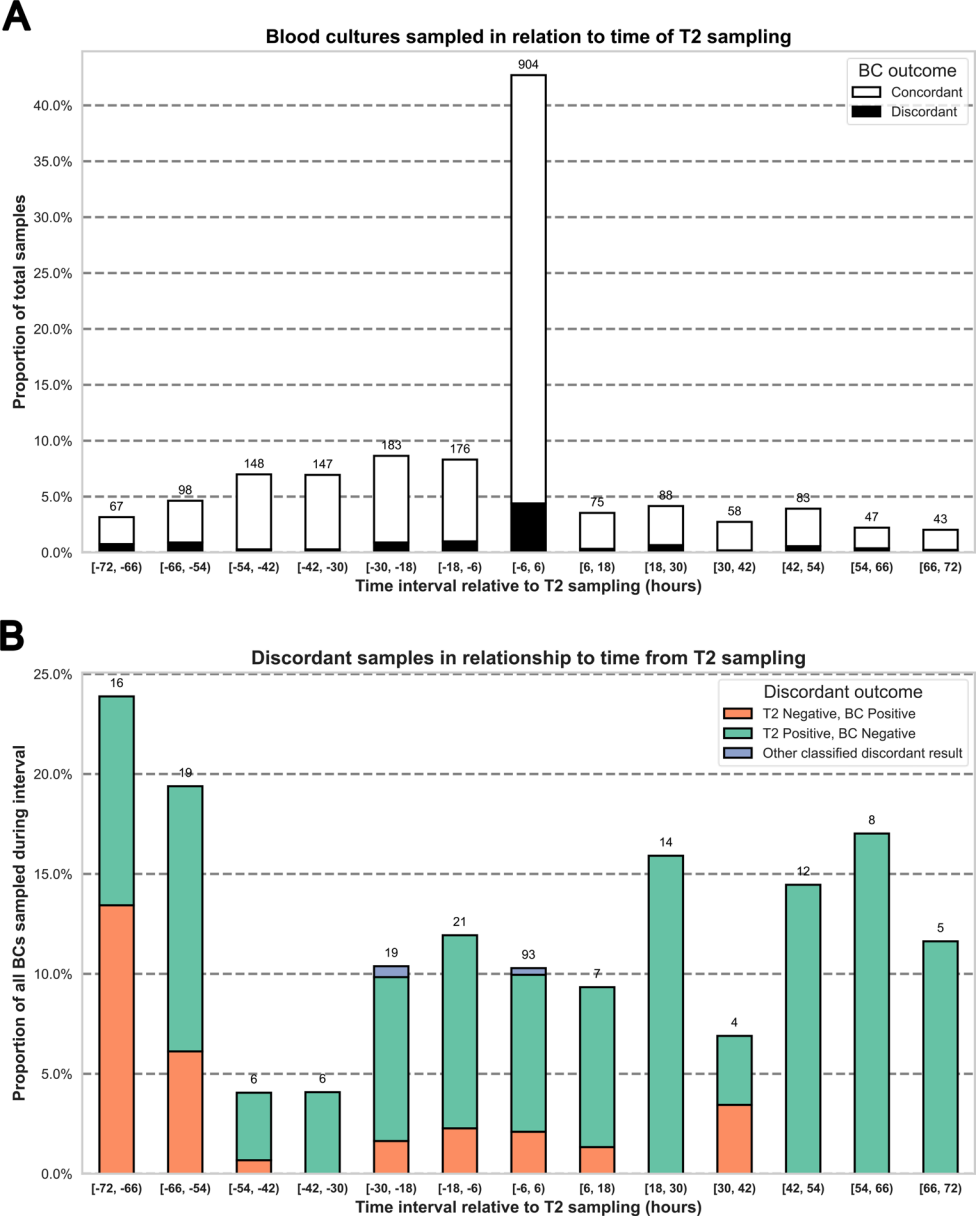
Time relationship between T2 and BC sampling. BC: Blood culture. T2: T2Bacteria. Total BC samples: n = 2,117. **Panel A**: BCs sampled in relation to T2 sampling time. Numbers above column bars denote total number of BCs sampled during the specified interval. **Panel B**: Discordant BCs in relation to T2 sampling time, shown as percentage of total BCs sampled during that time period (as shown in Panel A). Numbers above column bars denote absolute numbers. Four BCs were denoted as “Other classified discordant results”, of which three was BCs that detected one of two bacteria detected in T2, and one was a BC that detected two bacteria when T2 only detected one. Intervals are denoted by [start, end), where the square bracket ‘[‘ includes the initial value ‘start’, and the parenthesis ‘)’ excludes the terminal value ‘end’

Episode and sample characteristics are shown in Table 1. The mean (SD) patient age was 57.3 (19.8) years and 329/544 (60.5%) were male. There were 478 patients with one episode, 46 patients with two episodes, 13 patients with three episodes and seven patients with four episodes. The median (IQR) number of BCs associated with each T2 sample was 3 (2–4), and the median number of other microbiological samples was 1 (0–2). The mean (± SD) time difference between T2 and BC sampling was 21 (± 22.6) hours, and the distribution of BC samples with respect to T2 sampling is shown in Fig. 1, panel A.

**Table 1:** Characteristics of episodes and samples

Characteristic	Value
Episodes, *n* = 640	*n* (% of episodes)
Episodes where T2 sampling occurred in ICU	116 (18.1)
BC samples per episode	
- Episodes with 1 BC	115 (18.0)
- Episodes with 2 BCs	174 (27.2)
- Episodes with 3 BCs	133 (20.8)
- Episodes with 4 BCs	82 (12.8)
- Episodes with 5 BCs or more	134 (20.9)
Episodes with other microbiological samples taken	478 (74.7)
**Samples, ** ***n = 3876***	**n (% of samples)**
T2 samples	640 (16.5)
BC samples	2117 (54.6)
Non-BC microbiological samples	1119 (28.9)
- Urine	459 (41.0)*
- Lower respiratory tract	235 (21.0)*
- Deep wound/abscess/drainage	154 (13.8)*
- Superficial wound	105 (9.4)*
- Other sites	166 (14.8)*

BC: Blood culture. T2: T2Bacteria. ICU: Intensive care unit

*Percentages denote % of all non-BC microbiological samples

### Comparative analysis of T2 and BC considering in-panel bacteria

**Fig. 2:**
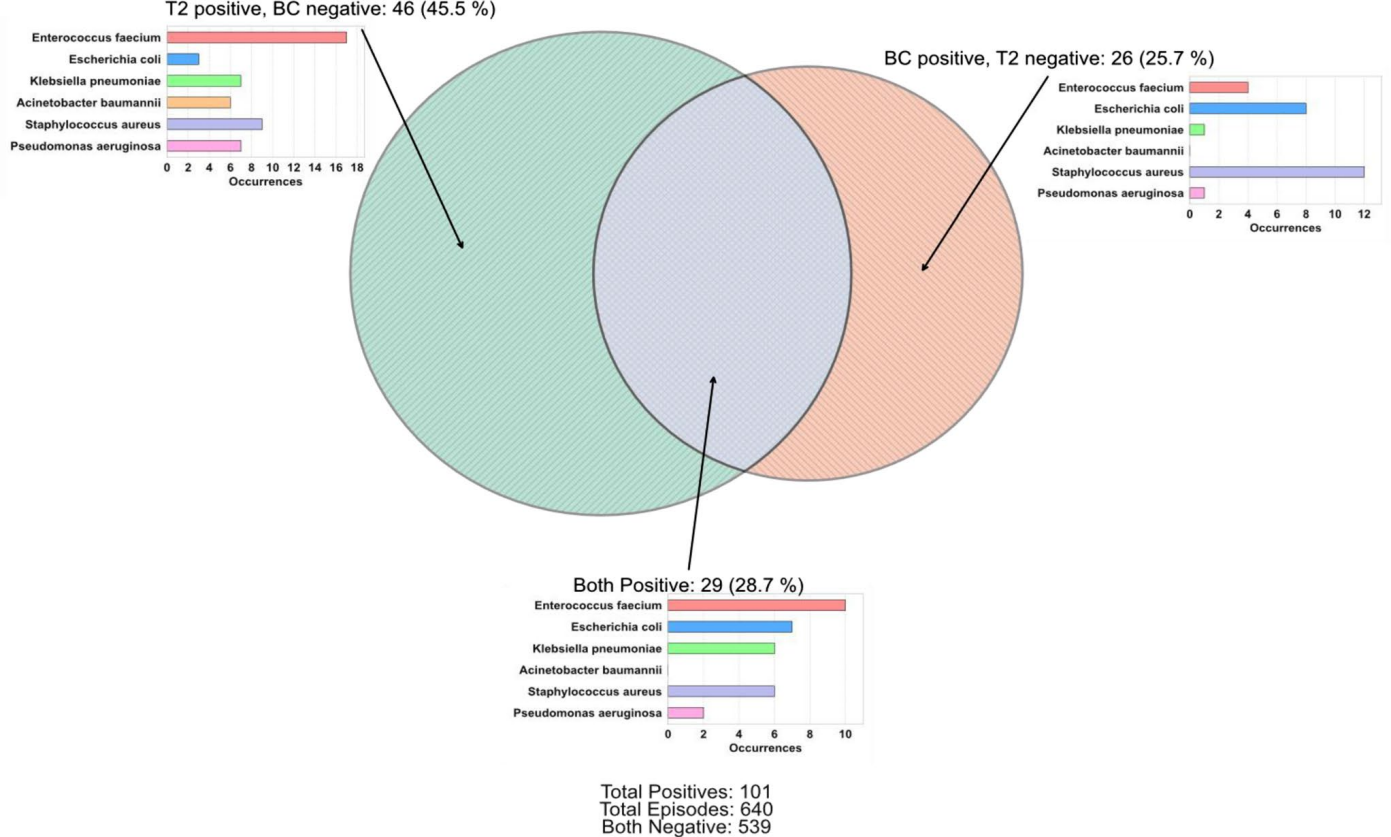
Venn diagram of characterization of episodes. BC: Blood culture. T2: T2Bacteria. Only in-panel bacteria are included in this analysis. Percentages denote % of total positives (*n* = 101). Episodes positive for both methods (*n* = 29) include 27 episodes in which isolate/isolates detected by T2 matched the isolate/isolates in BCs completely, while two episodes had partial matches

**Fig. 3:**
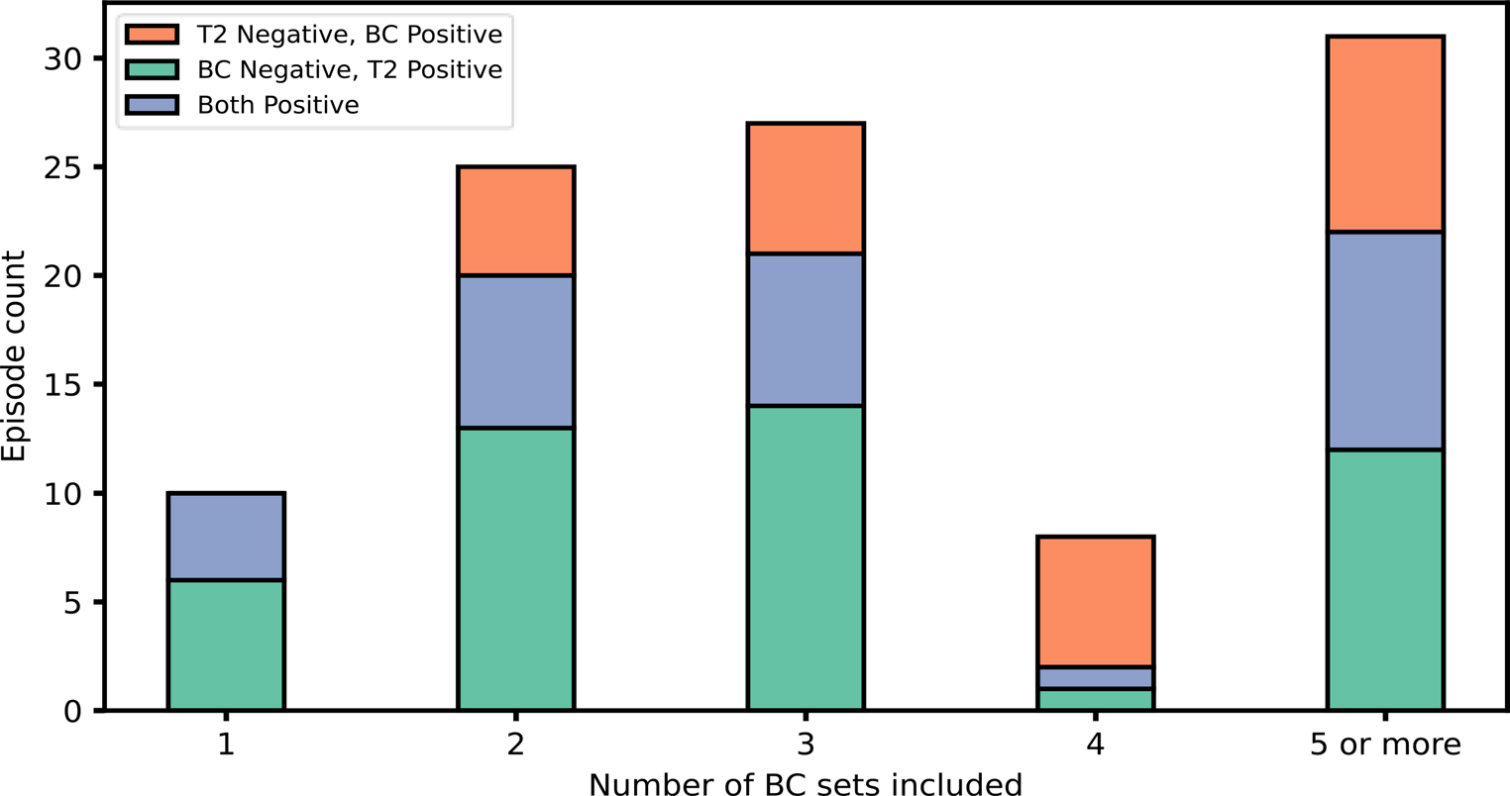
Episode classifications stratified by number of BC sets included in the analysis. BC: Blood culture. T2: T2Bacteria. Only episodes positive for in-panel bacteria are shown in the figure (*n* = 101)

Of the 640 episodes studied, 29 (4.5%) were T2 positive/BC positive, 46 (7.2%) were T2 positive/BC negative, 26 (4.1%) were T2 negative/BC positive, and 539 (84.2%) were negative with both methods. In total, 101 (15.8%) episodes were positive by either method, and the overlap of these episodes is shown in Fig. 2. For the 29 episodes that were positive for both methods, 27 episodes had identically matching T2 and BC results (considering in-panel bacteria). Of the two remaining episodes that were positive for both methods, one episode was T2 positive for *E. faecium* and *K. pneumoniae* while BC detected only *E. faecium*, and one episode was T2 positive for *E. faecium* while BC detected *E. faecium and S. aureus.* When comparing the discordant results of T2 positive/BC negative and T2 negative/BC positive episodes (46 and 26 respectively), there was a difference in frequencies (McNemar χ^2^, *p* = 0.018). Stratification by number of sampled BCs per episode is shown in Fig. 3. There were 4 episodes with polymicrobial T2 detections, and one episode that had polymicrobial growth in BCs. The sampling time of the BC samples that were discordant with T2 results are shown in Fig. 1, panel B. The discordant outcomes for T2 positive/BC negative and T2 negative/BC positive episodes are detailed in Supplement Table 2a and b, respectively.

### Evaluation of T2 positive/BC negative episodes

For episodes that were T2 positive/BC negative, T2 results were matched to non-BC microbiological samples to determine whether a T2 positive/BC negative episode represented a true positive or a possible false negative. For the 46 episodes that were T2 positive and BC negative, 35 had at least one other microbiological sample taken during the episode, of which eight (23%) had at least one isolate that matched the T2 result. If only these eight episodes were considered as true positives for T2, discordant pairs were significantly different in favor of BC, 46/640 vs. 8/640 episodes (McNemar χ^2^, *p* < 0.01).

For validation, we also determined the proportion in which T2 negative/BC positive episodes and T2 positive/BC positive episodes had a non-BC microbiological sample with growth of the same microorganism. For the 26 episodes that were T2 negative and BC positive, 22 had at least one other microbiological sample taken during the episode, of which 13 (59%) had at least one isolate that matched the BC result. For the 29 T2 positive/BC positive episodes, 20 had at least one other microbiological sample taken during the episode, of which 15 (75%) episodes had a matching non-BC sample.

### Analysis including microorganisms not included in the T2Bacteria panel

In a secondary analysis, all microorganisms in BCs were included to assess the impact of the limited repertoire imposed by the T2Bacteria panel. When considering all microorganisms as opposed to only in-panel microorganisms, the episodes that were classified as BC positive increased from 55 (8.6%) to 87 (13.6%). The complete list of detected microorganisms not included in the T2 panel is detailed in Supplement Table 3. Reclassifying episodes regarding BC positivity considering all detected microorganisms in BCs, T2 positive/BC negative episodes decreased from 46 to 42 and T2 negative/BC positive episodes increased from 26 to 54. In four episodes, T2 and BC had non-matching bacteria and in 6 polymicrobial episodes, the set of bacteria in T2 constituted a subset of bacteria found in BCs. Lastly, there was one episode where BC detected a subset of bacteria found in T2. The correlation of isolates in polymicrobial episodes is detailed in Table 2.

**Table 2:** Correlation of T2Bacteria and blood culture isolates in polymicrobial BSI episodes

Isolates detected by T2	Isolates detected by BC	Isolates detected by other microbiological samples
*Enterococcus faecium*	*Candida albicans* *Enterococcus faecium* Gram negative rod, unspecified* Gram positive coccus, unspecified* *Saccharomyces cerevisiae*	
*Enterococcus faecium* *Escherichia coli*		
	*Enterococcus faecium* *Neisseria* species *Streptococcus mitis/sanguinis* group	
*Escherichia coli*	*Enterococcus avium* *Escherichia coli*	*Candida tropicalis* (LRT)
*Klebsiella pneumoniae*	*Enterococcus faecalis* *Klebsiella pneumoniae*	*Klebsiella pneumoniae* (Urine)
	*Candida albicans* *Candida tropicalis* *Sphingomonas paucimobilis*	
*Enterococcus faecium Klebsiella pneumoniae*	*Enterococcus faecium*	
*Enterococcus faecium*	*Enterococcus faecium* *Fusarium* species	
	*Macrococcus* species *Staphylococcus aureus*	*Staphylococcus aureus* (SW)
	*Bacteroides fragilis* group *Candida albicans*	
*Pseudomonas aeruginosa*	*Citrobacter freundii* *Pseudomonas aeruginosa*	
*Enterococcus faecium* *Escherichia coli*		*Citrobacter freundii* (DwAD) *Enterococcus faecium* (DwAD) CoNS (VC)
*Pseudomonas aeruginosa*	*Sphingomonas paucimobilis* *Sphingomonas* species	*Corynebacterium* species (Misc) CoNS *(*VC) *Sphingomonas paucimobilis* (Misc) *Sphingomonas* species (VC)
*Enterococcus faecium* *Klebsiella pneumoniae*		
*Enterococcus faecium*	*Candida tropicalis*	
*Enterococcus faecium*	*Candida albicans*	Yeast, unspecified* (Urine)
*Enterococcus faecium*	*Enterococcus faecium* *Staphylococcus aureus*	
*Enterococcus faecium*	*Enterobacter cloacae* *Klebsiella oxytoca*	

Rows represent episodes that had polymicrobial findings considering the set of all isolates detected by T2 and BC.

The inclusion of isolates is irrespective of their presence in the T2Bacteria panel. Parentheses next to isolates in the last column indicate the respective sampling sites for additional microbiological specimens

BC: Blood culture. T2: T2Bacteria. CoNS: Coagulase negative staphylococci. LRT: Lower respiratory tract. SW: Superficial wound. DwAD: Deep wounds, abcesses and drainage. VC: Vascular catheter tip

*For these isolates, further identification was not possible

### Analysis of turn-around time

#### Comparison between BC and T2 turn-around times

**Fig. 4:**
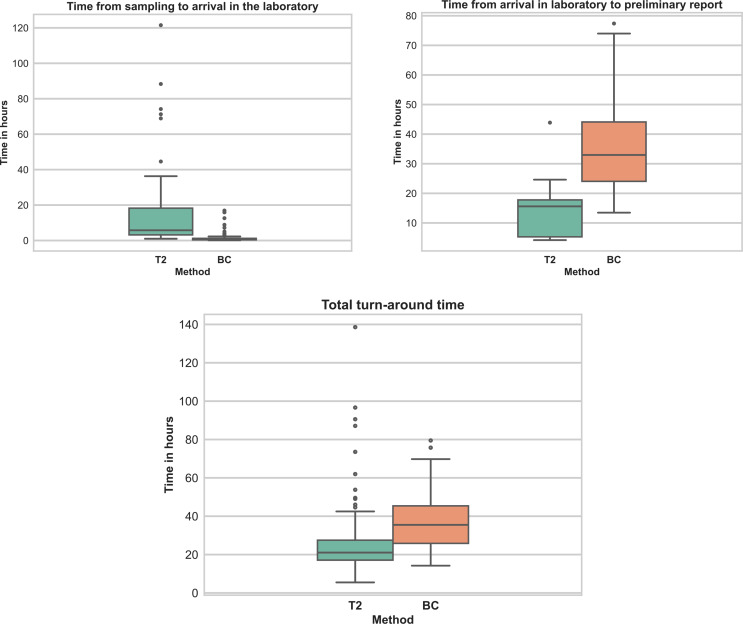
Turn-around time for T2Bacteria and blood culture samples. T2: T2Bacteria. BC: Blood culture. Only T2 and BC samples positive for bacteria included in the T2Bacteria assay panel (*n* = 75 for T2, *n* = 120 for BC) were included in this analysis. Total turn-around time is defined as the total time from sampling to preliminary report

TAT for in-panel positive BCs and T2 samples are shown in Fig. 4. The distribution for TAT was heavily right skewed and was described with median (IQR), and all comparisons were made using the Mann-Whitney U test. Total TAT from sampling to preliminary report for BC was 35 h and 30 min (25 h 50 min − 45 h 24 min), compared to 21 h and 3 min for T2 (17 h 6 min − 27 h 30 m) (*p* < 0.001). The time from sampling to arrival of the sample in the laboratory was 38 min for BC (23 min–1 h 13 min), and 5 h and 46 min for T2 (3 h 10 min − 18 h 16 min) (*p* < 0.001). The duration from the arrival of the sample to the first preliminary result was 32 h and 58 min for BC (24 h 3 min − 44 h 7 min) and 15 h and 36 min for T2 (5 h 18 min − 17 h 47 min) (*p* < 0.001) (Fig. 4).

#### T2 turn-around time for positive and negative samples

**Fig. 5:**
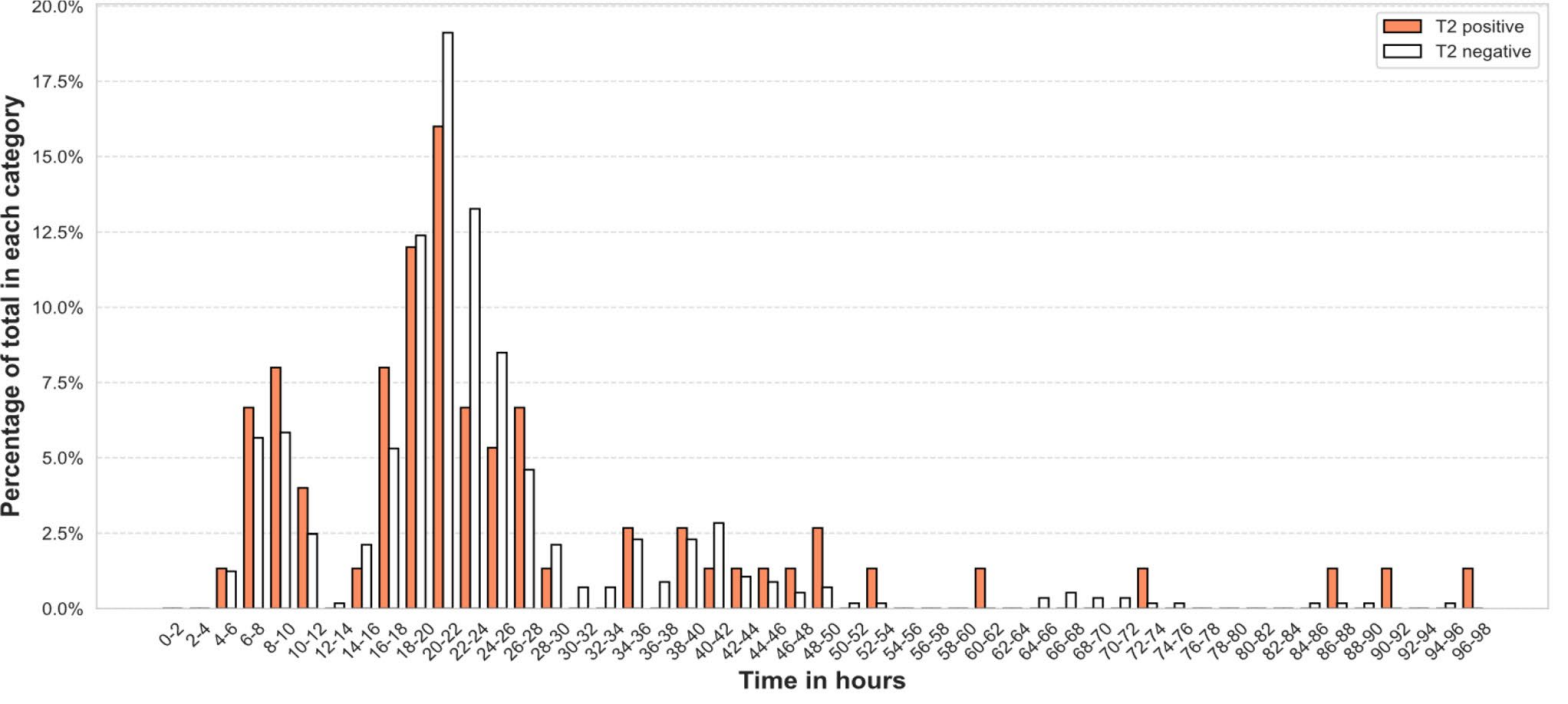
Distribution of turn-around times for positive and negative T2 samples. T2: T2Bacteria. Total *N* = 640

The distribution for TAT for positive and negative samples is shown in Fig. 5. TAT was 21 h and 3 min (17 h 6 min − 27 h 30 min) for positive samples and 21 h and 28 min (18 h 16 min − 25 h 36 min) for negative samples (*p* = 0.92).

#### T2 turn-around time by weekday and time of day

**Fig. 6:**
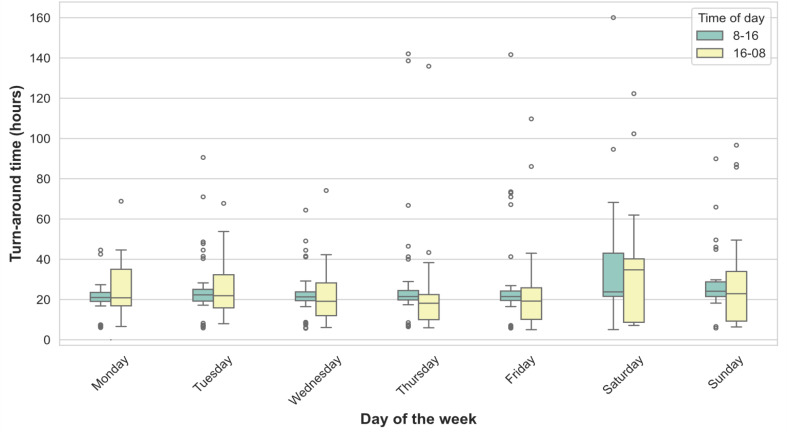
Turn-around time for T2Bacteria by weekday and time for sampling during the day. T2: T2Bacteria. Total *N* = 640. Boxplots are shown for T2 turnaround time based on day of the week and time of the day

The impact on T2 sample TAT by weekday and time of day for sampling is shown in Fig. 6. TAT was 21 h and 10 min (18 h 25 min to 25 h 6 min) for weekday (Monday – Friday) sampling and 23 h and 58 min (20 h 14 min to 39 h 30 min) for weekend sampling (*p* < 0.01). For time of day, TAT was 21 h 46 min (19 h 43 min − 25 h 2 min) for sampling that occurred 8 a.m. to 4 p.m., and 20 h 15 min (11 h 0 min − 32 h 20 min) for sampling occurring between 4 p.m. and 8 a.m. (*p* = 0.013).

## Discussion

In the present study, we assessed the performance of BCs and the T2Bacteria assay for the detection of bacterial pathogens in BSI, as well as TAT. Notably, the combined in-panel bacterial detection rate attributable to both methods was 15.8%, higher than bacteremia rates found in previous studies [9, 11]. Furthermore, T2 was positive in a higher proportion of cases than the BCs. Of particular interest is that most episodes - nearly three-quarters of all positive detections of in-panel bacteria - were characterized by discordant results where one test yielded a positive result while the other did not.

Almost half of all episodes were positive by T2 only, with no BCs sampled within the 72-hour window surrounding the T2 testing that were positive for the same isolate. For the episodes that had other non-BC microbiological samples for comparison, only 23% of the episodes had the same pathogen isolated from the other samples and could therefore be regarded as having a true positive T2 result.

As noted in previous studies [9, 11], finding a “gold standard” reference in T2 positive/BC negative cases is problematic, as there is no method of confirming that the test represents a true positive finding. This is demonstrated by the fact that, although higher than in the T2 positive/BC negative group, the proportion of episodes that had matching non-BC microbiological samples were only 59% in the BC positive/T2 negative group and 75% in the group with concordant BC and T2 results. This implies that the method of determining significance of isolated positive findings with T2 (or any novel BSI diagnostic method) used in this study as well as previous studies is not entirely reliable. Previous studies used a composite reference standard consisting of non-BC cultures and clinical adjudication. The latter, however, is influenced by the test itself which might lead to misclassification. The bacteria identified by T2 are among the most implicated in clinically significant infections and consequently, a positive T2 result, even in the absence of a supporting BC or a corresponding microbiological sample from another site, could indicate a true infection rather than a false positive. This observation is particularly crucial in settings in which prior antibiotic administration may have reduced the performance of BCs.

From the other viewpoint, around a quarter of all episodes were T2 negative but BC positive. The proportion of T2 negative/BC positive episodes are higher than reported in previous studies with a smaller sample size [11, 12, 14]. The reason for this difference is not obvious. One aspect is that most previous reports only included BCs sampled at the very same occasion as the T2 sampling. In addition, in several studies including the largest study by Nguyen et al. [9, 12], only one BC set was included as the comparator. It is well known that the practice of collecting only one BC set (e.g. solitary BC) limits the detection capabilities of BCs substantially, and a minimum of two BC sets are recommended in the guidelines [2, 18]. In contrast, the present study included all BCs during a time frame surrounding the T2 test, reaching a median number of three BCs per T2 test included. Therefore, the use of BCs as the comparator in the present study is used more closely in-line with the guidelines for BC sampling. This increases the diagnostic power of the comparator by both increasing the BC sampling volume and the number of BC sets. In our results, this was reflected by higher detection rate by BC in episodes with more BCs taken. Notably, in the 115 episodes that only included a single BC set, no additional diagnostic yield was achieved by BC.

When expanding the scope of detection to microorganisms not included in the T2 panel, BCs detected pathogens in additional 32 episodes, corresponding to a relative increase of 58%. This raises concerns about T2’s limited repertoire in clinical practice and underscores the need for a more comprehensive panel to capture a broader spectrum of bacterial infections as well as the complementary benefit of BCs. Polymicrobial BSI is increasingly common and ranges from 2 to 20% in current literature [19]. In the present study, the majority of polymicrobial BSIs included microorganisms not included in the T2 panel, suggesting that the limitation in detecting polymicrobial episodes is partly due to the limited repertoire with T2 bacteria. However, the polymicrobial sample size is small and warrants further study.

TAT is an important metric in determining the utility of a diagnostic method for BSI. As TAT for BCs still remains high due to the culture step, varying between 12 and 48 h for most cases [5, 12], molecular methods directly from blood have the potential to reduce TAT. As expected, we found that the total TAT was shorter with T2 than BC, however, the difference was partially balanced out by a substantial delay in the workflow for T2. Previous studies have shown a TAT around 3–7 h for T2 [9, 11, 12], however, providing only a portion of the actual TAT as the metric in these studies were defined as the sample processing time.

One concern has been that a longer TAT could negatively affect the performance of the T2 assay. However, this study shows that the TAT for positive and negative T2 samples did not significantly differ, providing reassurance against this concern. In most centers including the present study setting, T2 is a novel method and only available during work hours, which can lead to very long times to arrival. In addition, in settings centralized to one laboratory such as in the present study, delays imposed by geographic distances can be substantial. When examining how different sampling times affect TAT, the analysis revealed a significant difference between weekday and weekend sampling, as well as variations based on the time of day. This was attributed to a wider spread and more outliers during weekends and non-work hours. Importantly, median TAT was around 20 h even during weekday work-hour sampling.

### Limitations

The present study was a retrospective study, including only patients that underwent T2 sampling. Potentially, this approach might introduce a bias in patient selection as well as a limited panorama of studied infections. As this was a laboratory data study, we did not obtain comprehensive clinical data, and therefore the results are limited by lack of information about ongoing antibiotic use, indications for T2 sampling, and pertinent clinical data that could differentiate true from false positive results in the case of T2 positive/BC negative episodes. However, we used a similar approach as previous studies to assess the likelihood of true infection, considering additional microbiological samples in our assessment. The present study was performed at a single laboratory, limiting the generalizability of the data study and warrants caution when interpreting the results in an international context. However, the samples were collected from six large hospitals across Stockholm with a broad representation of patient categories, which in turn increases the external validity of the results.

### Strengths

There are several strengths to this study. Compared to prior studies of T2Bacteria [12–14, 20], the sample size is large, and the bacteremia rate is high. We have provided a conservative approach to the definition of positive BCs, taking the aggregate results of all BC samples within 72 h of T2 sampling as the comparative measure, with the majority of episodes including three or more BC sets. This served to ensure a proper BC sample volume and reduced the impact of potential intermittent bacteremia. In contrast to the controlled setting of “one T2 sample versus one BC sample” already studied in previous reports [9, 12], we have taken in account that clinical practice often includes several BC samplings across several time points during suspicion of infection. The TAT was for the first time described for T2Bacteria using an in-depth analysis, which reflect the actual sample-to-report time and not only the time for assay completion.

## Conclusions

The present study shows that T2Bacteria panel can be used as a potential adjunct to traditional BCs in the diagnosis of BSI. While each method has its inherent limitations, the combined use is promising to provide a comprehensive diagnostic strategy for BSIs. To improve TAT, it is crucial to optimize the workflow of emerging molecular BSI diagnostics.

## Electronic supplementary material

Below is the link to the electronic supplementary material.

Supplementary Material 1

Supplementary Material 2

## Data Availability

Data analysis methods are provided as open access (https://github.com/Dyu5mp8/T2Bact_study). The original dataset is available from the corresponding author upon reasonable request.

## References

[1] Evans L, Rhodes A, Alhazzani W, Antonelli M, Coopersmith CM, French C, Machado FR, McIntyre L, Ostermann M, Prescott HC, Schorr C, Simpson S, Wiersinga WJ, Alshamsi F, Angus DC, Arabi Y, Azevedo L, Beale R, Beilman G, Levy M (2021) Surviving sepsis campaign: international guidelines for management of sepsis and septic shock 2021. Intensive Care Med 47(11):1181–1247 34599691 10.1007/s00134-021-06506-y PMC8486643

[2] Lamy B, Dargere S, Arendrup MC, Parienti JJ, Tattevin P (2016) How to optimize the use of blood cultures for the diagnosis of Bloodstream infections? A state-of-the art. Front Microbiol 7:697 27242721 10.3389/fmicb.2016.00697 PMC4863885

[3] Banerjee R, Ozenci V, Patel R (2016) Individualized approaches are needed for optimized blood cultures. Clin Infect Dis 63(10):1332–1339 27558570 10.1093/cid/ciw573 PMC5091349

[4] Yu D, Unger D, Unge C, Parke A, Sunden-Cullberg J, Stralin K, Ozenci V (2022) Correlation of clinical sepsis definitions with microbiological characteristics in patients admitted through a sepsis alert system; a prospective cohort study. Ann Clin Microbiol Antimicrob 21(1):7 35193588 10.1186/s12941-022-00498-3 PMC8864844

[5] Peri AM, Harris PNA, Paterson DL (2022) Culture-independent detection systems for bloodstream infection. Clin Microbiol Infect 28(2):195–201 34687856 10.1016/j.cmi.2021.09.039

[6] Tassinari M, Zannoli S, Farabegoli P, Pedna MF, Pierro A, Mastroianni A, Fontan R, Luongo L, Sarnataro G, Menegatti E, Caruso A, Sambri V (2018) Rapid diagnosis of bloodstream infections in the critically ill: evaluation of the broad-range PCR/ESI-MS technology. PLoS ONE 13(5):e0197436 29763469 10.1371/journal.pone.0197436 PMC5953471

[7] Opota O, Jaton K, Greub G (2015) Microbial diagnosis of bloodstream infection: towards molecular diagnosis directly from blood. Clin Microbiol Infect 21(4):323–331 25686695 10.1016/j.cmi.2015.02.005

[8] Neely LA, Audeh M, Phung NA, Min M, Suchocki A, Plourde D, Blanco M, Demas V, Skewis LR, Anagnostou T, Coleman JJ, Wellman P, Mylonakis E, Lowery TJ (2013) T2 magnetic resonance enables nanoparticle-mediated rapid detection of candidemia in whole blood. Sci Transl Med 5(182):182ra154 10.1126/scitranslmed.3005377 23616121

[9] Nguyen MH, Clancy CJ, Pasculle AW, Pappas PG, Alangaden G, Pankey GA, Schmitt BH, Rasool A, Weinstein MP, Widen R, Hernandez DR, Wolk DM, Walsh TJ, Perfect JR, Wilson MN, Mylonakis E (2019) Performance of the T2Bacteria panel for Diagnosing Bloodstream infections: a diagnostic accuracy study. Ann Intern Med 170(12):845–852 31083728 10.7326/M18-2772

[10] Clancy CJ, Nguyen MH (2018) T2 magnetic resonance for the diagnosis of bloodstream infections: charting a path forward. J Antimicrob Chemother 73(suppl4):iv2–iv5 29608754 10.1093/jac/dky050

[11] De Angelis G, Posteraro B, De Carolis E, Menchinelli G, Franceschi F, Tumbarello M, De Pascale G, Spanu T, Sanguinetti M (2018) T2Bacteria magnetic resonance assay for the rapid detection of ESKAPEc pathogens directly in whole blood. J Antimicrob Chemother 73(suppl4):iv20–iv26 29608753 10.1093/jac/dky049

[12] Voigt C, Silbert S, Widen RH, Marturano JE, Lowery TJ, Ashcraft D, Pankey G (2020) The T2Bacteria assay is a sensitive and Rapid detector of Bacteremia that can be initiated in the Emergency Department and has potential to favorably influence subsequent therapy. J Emerg Med 58(5):785–796 31982197 10.1016/j.jemermed.2019.11.028

[13] Giacobbe DR, Crea F, Morici P, Magnasco L, Di Pilato V, Briano F, Willison E, Pincino R, Dettori S, Tutino S, Esposito S, Coppo E, Dentone C, Portunato F, Mikulska M, Robba C, Vena A, Battaglini D, Brunetti I, Bassetti M (2022) T2Bacteria and T2Resistance assays in critically ill patients with Sepsis or septic shock: a descriptive experience. Antibiot (Basel) 11:12 10.3390/antibiotics11121823 PMC9774778 36551480

[14] Quirino A, Scaglione V, Marascio N, Mazzitelli M, Garofalo E, Divenuto F, Serapide F, Bruni A, Lionello R, Pavia G, Costa C, Giancotti A, Peronace C, Longhini F, Russo A, Liberto MC, Matera G, Torti C, Trecarichi EM (2022) Role of the T2Dx magnetic resonance assay in patients with suspected bloodstream infection: a single-centre real-world experience. BMC Infect Dis 22(1):113 35105333 10.1186/s12879-022-07096-w PMC8805379

[15] (2022) Vårdplatser i Region Stockholm. Region Stockholm, https://www.regionstockholm.se/nyheter/2022/07/vardplatser-i-region-stockholm/. Cited July 21 2024

[16] Metzgar D, Frinder MW, Rothman RE, Peterson S, Carroll KC, Zhang SX, Avornu GD, Rounds MA, Carolan HE, Toleno DM, Moore D, Hall TA, Massire C, Richmond GS, Gutierrez JR, Sampath R, Ecker DJ, Blyn LB (2016) The IRIDICA BAC BSI assay: Rapid, sensitive and culture-independent identification of Bacteria and Candida in blood. PLoS ONE 11(7):e0158186 27384540 10.1371/journal.pone.0158186 PMC4934770

[17] Jordana-Lluch E, Rivaya B, Marco C, Gimenez M, Quesada MD, Escobedo A, Batlle M, Martro E, Ausina V (2017) Molecular diagnosis of bloodstream infections in onco-haematology patients with PCR/ESI-MS technology. J Infect 74(2):187–194 27889413 10.1016/j.jinf.2016.11.011

[18] Ekwall-Larson A, Yu D, Dinnétz P, Nordqvist H, Özenci V (2022) Single-site sampling versus Multisite Sampling for blood cultures: a Retrospective Clinical Study. J Clin Microbiol 60(2):e0193521 34851687 10.1128/JCM.01935-21 PMC8849186

[19] Fukushima S, Hagiya H, Fujita K, Kamiyama S, Yamada H, Kishida M, Otsuka F (2022) Clinical and microbiological characteristics of polymicrobial bacteremia: a retrospective, multicenter study. Infection 50(5):1233–1242 35301683 10.1007/s15010-022-01799-7

[20] Kalligeros M, Zacharioudakis IM, Tansarli GS, Tori K, Shehadeh F, Mylonakis E (2020) In-depth analysis of T2Bacteria positive results in patients with concurrent negative blood culture: a case series. BMC Infect Dis 20(1):326 32380973 10.1186/s12879-020-05049-9 PMC7206677

