# Supplementary material for: Performance of T2Bacteria in relationship to blood cultures - a retrospective comparative study
Source: Eur J Clin Microbiol Infect Dis. 2024 Aug 3;43(10):1977–87. doi: 10.1007/s10096-024-04916-6 (PMC11405434; doi:10.1007/s10096-024-04916-6)
Supplement: Supplementary file 1 — Supplementary Material 1 [file 10096_2024_4916_MOESM1_ESM.docx]

**Supplement table S1: Contaminant isolates**

| Bacteria |
| --- |
| Lactobacillus species |
| Cutibacterium (Propionibacterium) acnes |
| Staphylococcus epidermidis |
| Micrococcus species |
| Coagulase negative staphylococcus (CoNS) |
| Gram positive rod, unspecified |
| Bacillus cereus |
| Corynebacterium amycolatum |
| Corynebacterium species |
| Corynebacterium striatum |
| Aerococcus viridans |

**Supplement table S2a: T2 positive, BC negative episodes for in panel bacteria**

| T2 isolates | BC samples (n) | BC isolates | Non-BC cultures (n) | Non-BC cultures isolates |
| --- | --- | --- | --- | --- |
| Escherichia coli, Enterococcus faecium | 5 |  | 0 |  |
| Klebsiella pneumoniae | 2 |  | 0 |  |
| Staphylococcus aureus | 3 |  | 2 | Enterococcus faecium, Yeast, unspecified |
| Enterococcus faecium | 2 |  | 2 |  |
| Enterococcus faecium | 3 | Staphylococcus epidermidis | 3 |  |
| Klebsiella pneumoniae | 2 |  | 4 |  |
| Acinetobacter baumannii | 3 |  | 1 | Escherichia coli |
| Enterococcus faecium | 2 |  | 0 |  |
| Klebsiella pneumoniae | 5 |  | 0 |  |
| Staphylococcus aureus | 1 |  | 3 | **Staphylococcus aureus** |
| Staphylococcus aureus | 1 |  | 0 |  |
| Staphylococcus aureus | 3 |  | 2 | Yeast, unspecified |
| Pseudomonas aeruginosa | 3 |  | 1 |  |
| Escherichia coli | 3 |  | 1 |  |
| Staphylococcus aureus | 1 |  | 0 |  |
| Acinetobacter baumannii | 3 |  | 2 | Mixed flora |
| Acinetobacter baumannii | 2 |  | 0 |  |
| Enterococcus faecium | 8 | Coagulase negative staphylococci (CoNS) | 1 |  |
| Enterococcus faecium | 3 |  | 1 |  |
| Klebsiella pneumoniae | 3 |  | 1 |  |
| Staphylococcus aureus | 1 |  | 0 |  |
| Staphylococcus aureus | 2 |  | 2 |  |
| Staphylococcus aureus | 5 |  | 1 |  |
| Acinetobacter baumannii | 5 |  | 2 |  |
| Pseudomonas aeruginosa | 2 |  | 0 |  |
| Enterococcus faecium | 7 |  | 3 |  |
| Escherichia coli, Enterococcus faecium | 3 |  | 4 | Citrobacter freundii ESBL, Coagulase negative staphylococci (CoNS), **Enterococcus faecium** |
| Pseudomonas aeruginosa | 6 | Sphingomonas paucimobilis, Sphingomonas species | 2 | Corynebacterium species, Sphingomonas paucimobilis, Sphingomonas species, Coagulase negative staphylococci (CoNS) |
| Acinetobacter baumannii | 3 | Coagulase negative staphylococci (CoNS), Staphylococcus epidermidis | 3 | Pseudomonas aeruginosa |
| Enterococcus faecium | 2 |  | 4 | Mixed flora |
| Klebsiella pneumoniae, Enterococcus faecium | 2 |  | 0 |  |
| Acinetobacter baumannii | 1 | Coagulase negative staphylococci (CoNS) | 2 | Enterococcus faecalis, Staphylococcus aureus |
| Pseudomonas aeruginosa | 5 |  | 1 |  |
| Pseudomonas aeruginosa | 2 |  | 2 | **Pseudomonas aeruginosa,** Enterococcus faecium |
| Klebsiella pneumoniae | 6 |  | 3 | Escherichia coli, Gram-negative rod, Enterobacterales, Staphylococcus aureus |
| Enterococcus faecium | 5 |  | 3 | **Enterococcus faecium,** Yeast, unspecified |
| Enterococcus faecium | 8 | Candida tropicalis | 2 |  |
| Enterococcus faecium | 6 |  | 3 | **Enterococcus faecium,** Yeast, unspecified |
| Enterococcus faecium | 3 |  | 1 |  |
| Enterococcus faecium | 4 | Candida albicans | 4 | Yeast, unspecified |
| Enterococcus faecium | 2 |  | 1 | Enterobacter species, **Enterococcus faecium** |
| Staphylococcus aureus | 2 |  | 2 | **Staphylococcus aureus,** Group G streptococci |
| Pseudomonas aeruginosa | 3 |  | 8 | **Pseudomonas aeruginosa,** Group A streptococci (Streptococcus pyogenes) |
| Klebsiella pneumoniae | 1 |  | 2 |  |
| Pseudomonas aeruginosa | 3 |  | 0 |  |
| Enterococcus faecium | 2 | Klebsiella oxytoca, Enterobacter cloacae | 1 |  |

BC: Blood culture. T2: T2Bacteria. Matching isolates between T2 and non-BC cultures are shown in bold.

**Supplement table S2b: T2 negative, BC positive episodes for in panel bacteria**

| T2 isolates | BC samples (n) | BC isolates | Non-BC cultures (n) | Non-BC cultures isolates |
| --- | --- | --- | --- | --- |
|  | 2 | **Escherichia coli** | 1 |  |
|  | 3 | **Escherichia coli** | 1 | **Escherichia coli** |
|  | 2 | Streptococcus mitis/sanguinis, Neisseria species, **Enterococcus faecium,** Staphylococcus epidermidis | 1 |  |
|  | 3 | **Escherichia coli** | 1 |  |
|  | 4 | **Klebsiella pneumoniae** | 1 |  |
|  | 4 | **Enterococcus faecium,** Staphylococcus epidermidis | 0 |  |
|  | 7 | **Staphylococcus aureus** | 1 |  |
|  | 5 | **Staphylococcus aureus** | 0 |  |
|  | 5 | **Staphylococcus aureus** | 5 | **Staphylococcus aureus** |
|  | 4 | Coagulase negative staphylococci (CoNS), **Enterococcus faecium,** Staphylococcus epidermidis | 2 | Enterococcus faecium, Staphylococcus epidermidis |
|  | 2 | **Escherichia coli** | 1 | **Escherichia coli** |
|  | 2 | **Enterococcus faecium** | 0 |  |
|  | 4 | **Escherichia coli** | 2 | **Escherichia coli** |
|  | 3 | **Escherichia coli** | 2 | **Escherichia coli, Staphylococcus aureus** |
|  | 4 | Aerococcus viridans, Macrococcus species, Coagulase negative staphylococci (CoNS), **Staphylococcus aureus** | 1 | **Staphylococcus aureus** |
|  | 6 | **Pseudomonas aeruginosa** | 2 | **Staphylococcus aureus** |
|  | 8 | **Staphylococcus aureus** | 1 | **Staphylococcus aureus** |
|  | 6 | **Staphylococcus aureus** | 4 |  |
|  | 2 | **Staphylococcus aureus** | 4 | Gram-negative rod, Enterobacterales, **Staphylococcus aureus** |
|  | 6 | **Staphylococcus aureus** | 5 | **Staphylococcus aureus** |
|  | 4 | **Staphylococcus aureus** | 0 |  |
|  | 13 | **Staphylococcus aureus**, Coagulase negative staphylococci (CoNS) | 4 |  |
|  | 3 | Staphylococcus epidermidis, **Staphylococcus aureus** | 1 |  |
|  | 3 | **Staphylococcus aureus** | 1 | **Staphylococcus aureus**, Streptococcus pneumoniae |
|  | 5 | **Escherichia coli** | 2 | **Escherichia coli** |
|  | 3 | **Escherichia coli** | 2 | **Escherichia coli**, Coagulase negative staphylococci (CoNS) |

BC: Blood culture. T2: T2Bacteria. Matching isolates between BC and non-BC cultures are shown in bold.

**Supplement table S3: Episode count by bacteria not included in the T2Bacteria panel**

| Bacterial Species | Episode Count |
| --- | --- |
| Candida albicans | 8 |
| Streptococcus mitis/sanguinis group | 3 |
| Enterococcus faecalis | 3 |
| Gram-negative rod, unidentified | 2 |
| Stenotrophomonas maltophilia | 2 |
| Candida tropicalis | 2 |
| Sphingomonas paucimobilis | 2 |
| Streptococcus pneumoniae | 2 |
| Candida glabrata | 2 |
| Acinetobacter non-baumannii | 1 |
| Saccharomyces cerevisiae | 1 |
| Gram-positive cocci, unidentified | 1 |
| Neisseria species | 1 |
| Enterococcus avium | 1 |
| Achromobacter xylosoxidans | 1 |
| Fusarium species | 1 |
| Klebsiella (Enterobacter) aerogenes | 1 |
| Fusobacterium species | 1 |
| Haemophilus species | 1 |
| Macrococcus species | 1 |
| Bacteroides fragilis group | 1 |
| Citrobacter freundii | 1 |
| Sphingomonas species | 1 |
| Gemella species | 1 |
| Salmonella typhi | 1 |
| Candida pelliculosa (syn. Hansenula anomala) | 1 |
| Beta-hemolytic streptococcus group G | 1 |
| Group B streptococcus (Streptococcus agalactiae) | 1 |
| Beta-hemolytic streptococcus group A (Streptococcus pyogenes) | 1 |
| Klebsiella oxytoca | 1 |
| Enterobacter cloacae | 1 |
