# Supplementary material for: Performance of T2Bacteria in relationship to blood cultures - a retrospective comparative study
Source: Eur J Clin Microbiol Infect Dis. 2024 Aug 3;43(10):1977–87. doi: 10.1007/s10096-024-04916-6 (PMC11405434; doi:10.1007/s10096-024-04916-6)
Supplement: Supplementary file 2 — Supplementary Material 2 [file 10096_2024_4916_MOESM2_ESM.docx]

**
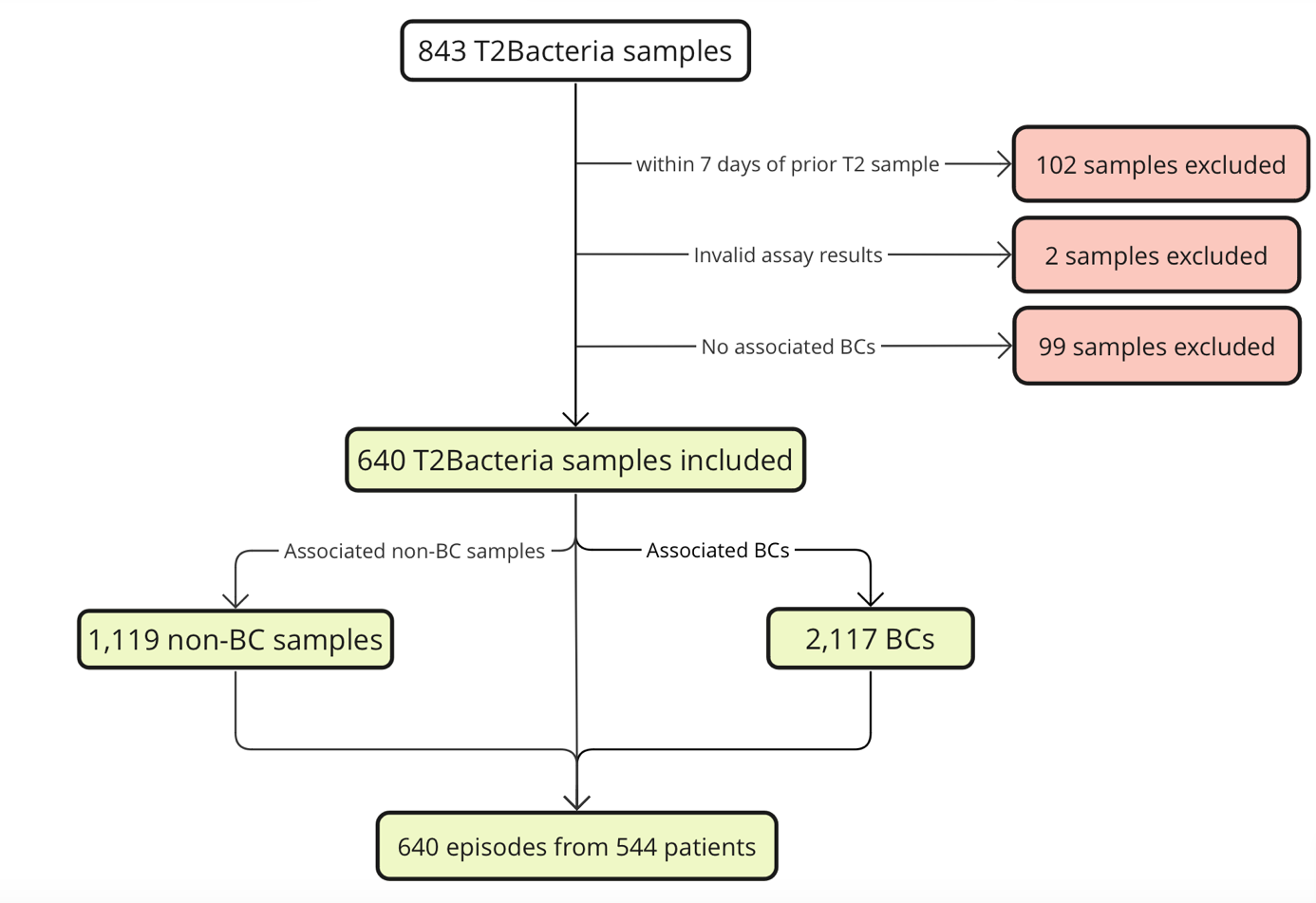
**

**Supplement figure 1:** **Study inclusion flow chart.** BC: Blood culture.

**Supplement figure 2:** **Numbers and proportions of concordant and discordant episodes as a function of the time frame used in the analyses.** T2: T2Bacteria. BC: Blood culture.
